# Short-Term Internet-Search Training Is Associated with Increased Fractional Anisotropy in the Superior Longitudinal Fasciculus in the Parietal Lobe

**DOI:** 10.3389/fnins.2017.00372

**Published:** 2017-06-29

**Authors:** Guangheng Dong, Hui Li, Marc N. Potenza

**Affiliations:** ^1^Department of Psychology, Zhejiang Normal UniversityJinhua, China; ^2^Department of Psychiatry, Department of Neurobiology, Yale School of Medicine, Yale UniversityNew Haven, CT, United States; ^3^Child Study Center, Yale School of Medicine, Yale UniversityNew Haven, CT, United States; ^4^CASAColumbia, Yale School of Medicine, Yale UniversityNew Haven, CT, United States

**Keywords:** internet search using, superior longitudinal fasciculus, DTI, spatial working memory

## Abstract

The Internet search engine has become an indispensable tool for many people, yet the ways in which Internet searching may alter brain structure and function is poorly understood. In this study, we investigated the influence of short-term Internet-search “training” on white matter microstructure using diffusion tensor imaging (DTI). Fifty-nine valid subjects (Experimental group, 43; Control group, 16) completed the whole procedure: pre- DTI scan, 6-day's training and post- DTI scan. Using track-based spatial statistics, we found increased fractional anisotropy in the right superior longitudinal fasciculus at post-test as compared to pre-test in experimental group. Within the identified region of the right superior longitudinal fasciculus, decreased radial diffusivity (RD), and unchanged axial diffusivity (AD) were observed. These results suggest that short-term Internet-search training may increase white-matter integrity in the right superior longitudinal fasciculus. A possible mechanism for the observed FA change may involve increased myelination after training, although this possibility warrants additional investigation.

## Introduction

The brain both promotes behavior and is modified by behaviors it promotes (Zatorre et al., 2012). In the past few decades, Internet search engines have become widely used Compared to other sources, the Internet may provide more immediate, reliable, and inexpensive access to information. However, the daily use of Internet searching may change our ability to process information (Dong and Potenza, 2016; Wang et al., 2017). Studies have revealed some cognitive changes related to Internet searching. Sparrow et al. found that people using Internet search engines became better at remembering where to find information, relying on the Internet to store the actual information (Sparrow et al., 2011). Searching for information online made people mistakenly think they have more knowledge, as indexed by an increase in self-assessed knowledge (Fisher et al., 2015). However, people showed less confidence in recalling information learned through Internet searching, with recent Internet searching possibly promoting motivations to continue to use the Internet for searching (Dong and Potenza, 2015). A short-term training (5 days) study involving older adults reported that Internet searching was related to brain responsiveness in neural circuits involved in decision-making and complex reasoning (Small et al., 2009).

Despite accumulating behavioral and fMRI work, knowledge about the neural mechanisms underlying training effects relating to Internet usage is scarce (Dong and Potenza, 2016; Wang et al., 2017), especially with respect to relationships between Internet searching and brain structure. In addition, cross-sectional studies do not provide insight into possible causal relations. As such, longitudinal studies are needed to explore possible effects of Internet searching on brain structure. Thus, this study sought to explore the possible effects of short-term Internet-search training on brain white-matter structures using diffusion tensor imaging (DTI).

DTI is a non-invasive MRI-based technique that can characterize micro-structural white matter changes by mapping molecular diffusion in biological tissues (Basser, 1995; Beaulieu, 2002). In DTI, the diffusion tensor can be computed and the principal direction of the diffusion tensor is used to infer white-matter connectivity. Fractional anisotropy (FA) is an important DTI metric that indicates the extent to which diffusion is non-random, and higher FA values have been proposed to reflect better “white-matter integrity” and has been associated with better task performance in certain instances (Tang et al., 2010; Bosch et al., 2012). Axial diffusivity (AD) and radial diffusivity (RD) are also important DTI indices. Alterations in AD may reflect axonal morphological changes (for example, relating to axonal density), and alterations in RD may reflect characteristics relating to myelination (for example, a decrease in RD may reflect increased myelination, and vice versa; Bennett et al., 2010; Kumar et al., 2012; Tang et al., 2012). By analyzing FA conjointly with RD and AD, it is possible to distinguish between diffusivity patterns that may reflect different neurobiological features (Pierpaoli et al., 1996; Itoh et al., 2000; Mukherjee et al., 2002; Thurnher et al., 2005).

Short-term training may modify brain white-matter structure, pointing to a rapid timescale of structural plasticity (Hill, 2013). For example, 2 h of training on a car-racing game training induces changes in the hippocampus and parahippocampal gyri (Sagi et al., 2012). Two hours of training on a spatial learning task in humans and 1 day's training on a Morris water maze in rats induces FA changes in the fornix (Hofstetter et al., 2013). An 11-h meditation training can alter white-matter connectivity in the anterior cingulate cortex (Tang et al., 2010, 2012). Ten weeks of memory training has been related to increased FA in anterior brain regions (Engvig et al., 2012). All of these results provide evidence for short-term white-matter plasticity in the human (and rat) brain, which suggests that the white matter in adults preserves dynamic characteristics and may be modified by short-term learning/training experiences. The daily use of Internet searching may be considered a type of “training,” and it might affect features of white matter involved in functionally connecting specific brain regions. Thus, we expected to find DTI changes associated with short-term Internet searching.

People who use Internet-based searches appear better at remembering the location of information rather than the information itself (Sparrow et al., 2011). Thus, we first hypothesized that Internet searching might increase white-matter connectivity in regions responsible for spatial memory, especially in the parietal lobe. Our previous studies showed that people performing Internet searches engaged less brain activations in declarative-memory-related brain regions along the ventral stream (Dong and Potenza, 2015, 2016). Thus, we hypothesized that changes might be observed in white matters connecting related brain regions such as the bilateral superior longitudinal fasciculus, which connects the parietal lobe with frontal, occipital and temporal lobes (Lebel et al., 2008); In addition, the temporal gyrus has been shown to be involved in spatial working memory (Karlsgodt et al., 2008; Shinoura et al., 2009; Vestergaard et al., 2011). We also hypothesized that the inferior longitudinal fasciculus, which connects the temporal lobe and occipital lobe and has been implicated in declarative memory, would be identified. We expected to observe Internet searching to relate to DTI measures with: (1) increased connections (e.g., as reflected by higher FA values) in spatial-working-memory-related brain structures; and (2) decreased connections (e.g., as reflected by lower FA values) in declarative-memory-related brain structures.

## Methods and procedures

### Participants

The experiment conforms to The Code of Ethics of the World Medical Association (Declaration of Helsinki). The Human Investigations Committee of Zhejiang Normal University approved this research. Sixty-six participants who were university students were recruited through advertisements (48 experimental group, 18 control group), 59 of whom completed the whole study (43 experimental group (male 23; female 20; age: 21.4 ± 1.2 years), 16 control group (male 7, female 9; age: 21.2 ± 0.5 years); No group difference was observed in age (*t* = 0.362, *p* = 0.639). All participants provided written informed consent and underwent structured psychiatric interviews (using the MINI; Lecrubier et al., 1997) performed by an experienced psychiatrist. All participants were free of psychiatric disorders (including major depression, anxiety disorders, schizophrenia, and substance dependence disorders) as assessed by the MINI. All participants were medication-free and were instructed not to use any substances, including coffee, on the day of scanning.

As it is impossible to find a person without experience of internet search using in university students. All subjects were assessed using an Internet-search questionnaire (Wang et al., 2016). The results of the questionnaire showed that all subjects were familiar with Internet searches and used the Internet regularly for such purposes. In addition, although all subjects has the experience of using Internet search, however, they just use it when necessary, nobody use it in an intense way like what we asked them to do in our “training” process.

Participants would be arranged into one of two groups randomly. Given financial limitations, we used the least number of healthy controls (16 valid subjects, which has already reached the request of neuroimaging studies) in current study; at the same time, we tried to recruit as more subjects as possible for experimental group.

### Training tasks

The experiment consisted of three steps: pre-training DTI scans, 6 days of “training,” and post-training DTI scans. The task was described as follows. In the current study, subjects were “trained” for about one and a half hours per day over a period of 6 consecutive days. During these 6 days, subjects were asked to finish one of six search tasks, randomly and with no repetition. Each search task consisted of 80 fill-in-the-blank items that required subjects to seek answers through using an Internet search engine. Participants were paid up to 20 Chinese Yuan per day for their participation. To increase their motivation in searching, subjects were informed that they would be paid according to their performance [20 ^*^ accuracy rates (%)].

In the control group, subjects were asked to use the computer for about 1.5 h per day. No limitations (except doing online searches) were presented to their online behaviors except doing one thing all the time during these six training periods (do one thing systematically); for example, read news or play online games.

### Imaging acquisition

DTI data were acquired with a 3.0T Siemens Trio scanner. Diffusion sensitizing gradients were applied along 64 non-collinear directions using *b*-value image 0 (1 b0 image) and 1,000 s/mm2 (TR = 6,800 ms, TE = 93 ms, matrix = 128^*^128, FOV = 256 mm^*^256 mm), with 50 contiguous slices acquired interleaved, and each slice was 2.5 mm thick. The entire scan lasted 10 min.

### Data processing

Preprocessing and data analysis were performed using PANDA (http://www.nitrc.org/projects/panda; Cui et al., 2013), a pipeline software based on the FSL diffusion toolbox (FDT, www.fmrib.ox.ac.uk/fsl/fdt; Smith et al., 2007). We used standard steps as proposed by FSL'S FDT, and these steps are described briefly as follows. The diffusion-weighted images were preprocessed by aligning to the *b* = 0 image using linear image registration for motion correction. A simple least-squares fit of the tensor model and diffusion tensor made use of the five *b*-values, and FA, as well as tensor eigenvalues that describe λ_1_ (represents the diffusion along the direction of the fibers) and λ_23_ (represents diffusion perpendicular to the fiber tract axis) were then calculated. The diffusion at each voxel was modeled with a Bayesian approach that produced a probability distribution along the principle diffusion direction later used for probabilistic tractography. The mean FA (also λ_1_ and λ_23_) image and its skeleton were next created from all subjects in MNI152 space. Then, each subject's FA map was projected onto the skeleton and voxel-wise statistics were performed across subjects for skeletonized FA images. An FA threshold of 0.2 was set to differentiate between white- and gray-matter in FSL (Smith et al., 2006). Finally, voxel-wise statistics of FA were conducted to test for regional differences in diffusion-related measures between pre- and post-tests. Finally, voxel-wise repeated-measures ANOVAs (group^*^ pre-post) were administered at each voxel using the randomize procedure in FSL with 5,000 permutations. Corrections for multiple comparisons were conducted using a threshold-free cluster enhancement method with a family-wise-error (FWE) threshold of *p* < 0.05.

## Results

**Figure 1:**
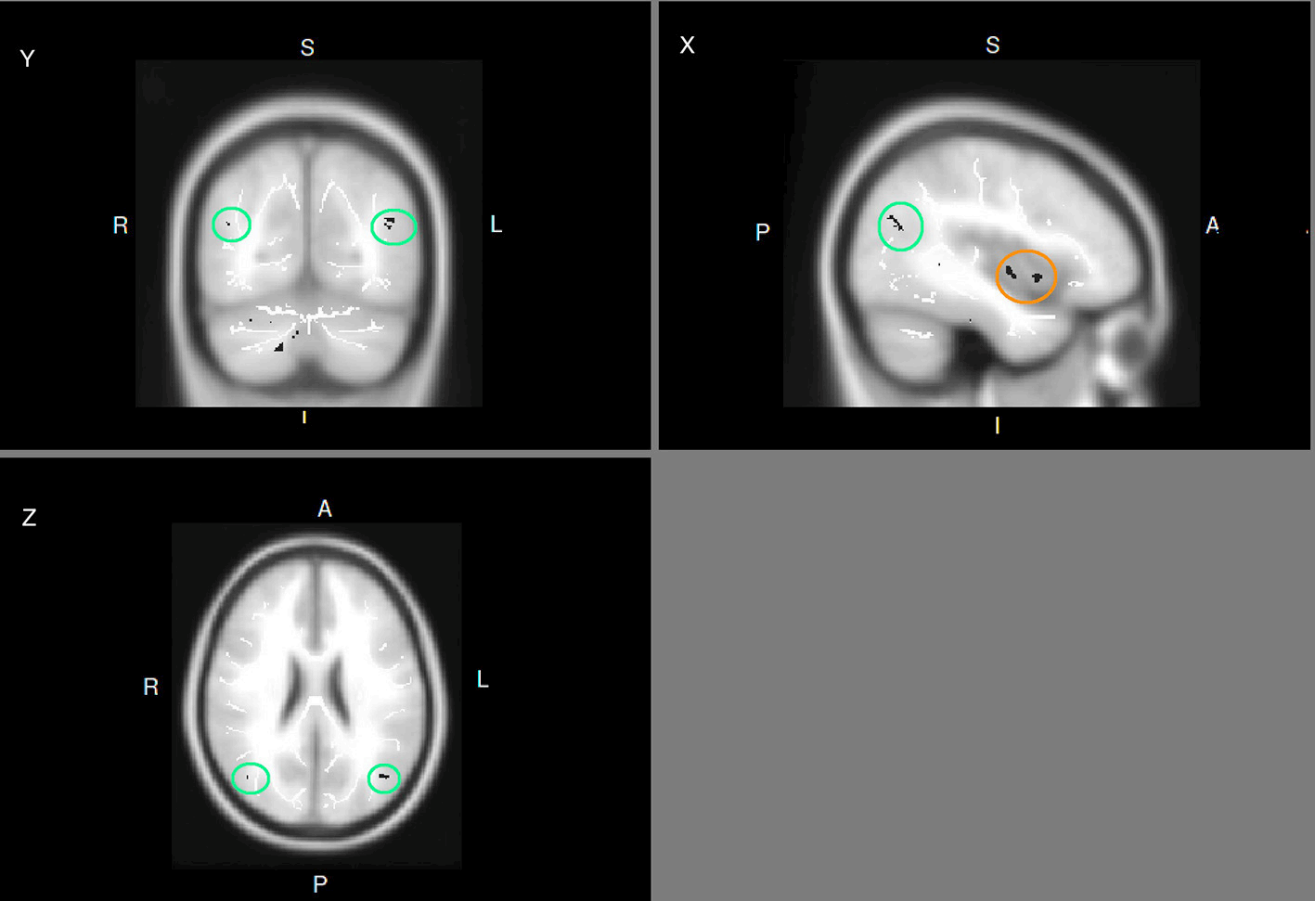
FA differences in post-training (experimental group minus control group) (x, y, z: −41, −71, 24). Areas in brown circle: superior temporal gyrus; Areas in green circle: bilateral parietal lobe.

The group comparison results showed that no brain regions show group difference in pre-test (*p* < 0.05). In post-test, the training group was associated higher FA changes in bilateral parietal lobe and right superior temporal lobe (Figure 1).

Further analysis paid attention to the post-pre difference in experimental and control groups, separately. Post-pre analysis showed that no measurable difference was found in the control group when comparing the post-test to pre-test DTI data (using *p* < 0.05). All the difference was caused by the FA changes in experimental group between pre- and post-tests. However, no difference was observed in declarative memory related brain structures, as we observed in post-training group comparison (right superior temporal lobe).

### Whole-brain FA changes from pre-training to post-training (post–pre)

**Figure 2:**
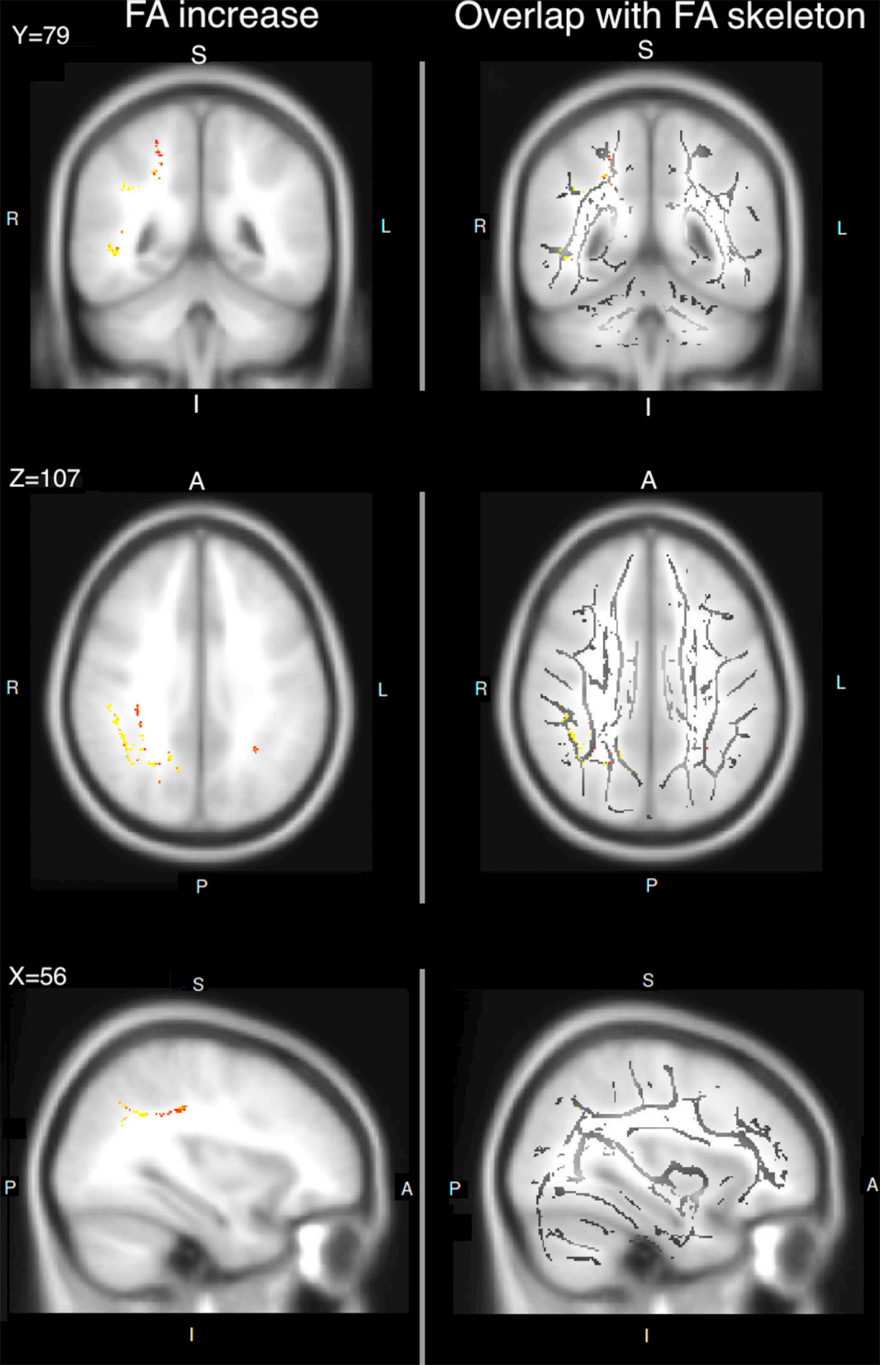
FA increases from pre- to post-training (post-pre) in experimental group. **(Left)** Statistical images of the FA changes; **(Right)** The statistical images are shown on the FA skeleton.

For the experimental group, we first performed whole-brain comparisons to examine all brain areas showing FA changes from pre-training to post-training. Multiple areas showed greater FA when comparing post-training to pre-training DTI measures (Figure 1). Most regions showing increased FA were located in the right superior longitudinal fasciculus in the parietal lobe (Figure 2). No decreases in FA were found post-training as compared to pre-training.

**Figure 3:**
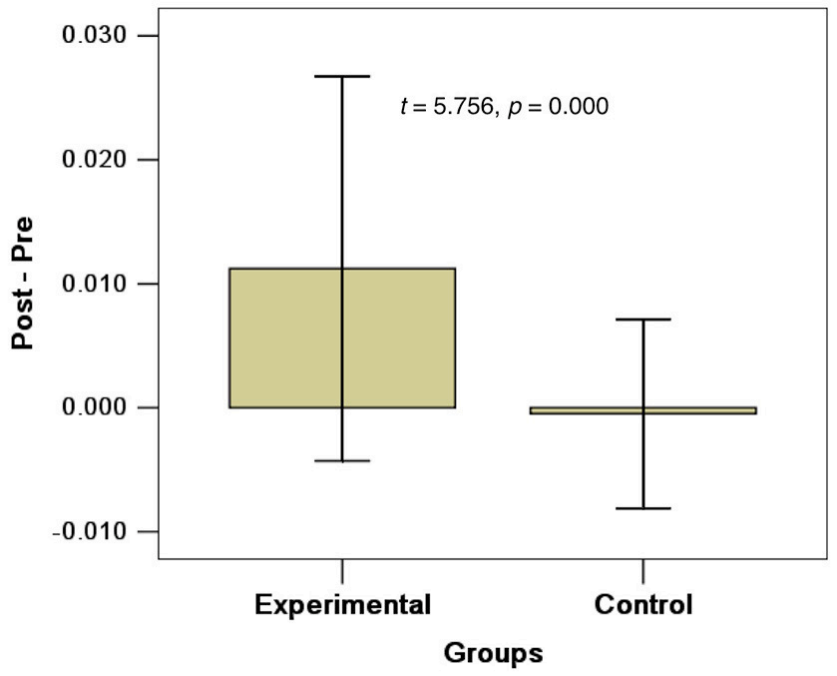
FA changes in post-pre in different groups (Mean ± *SD*).

The post-pre FA changes in different groups could be observed in Figure 3. From this figure, we can see the change in experimental group is much bigger than that in the control group. There are only slightly (maybe no) changes in the control group.

### ROI comparisons

**Figure 4:**
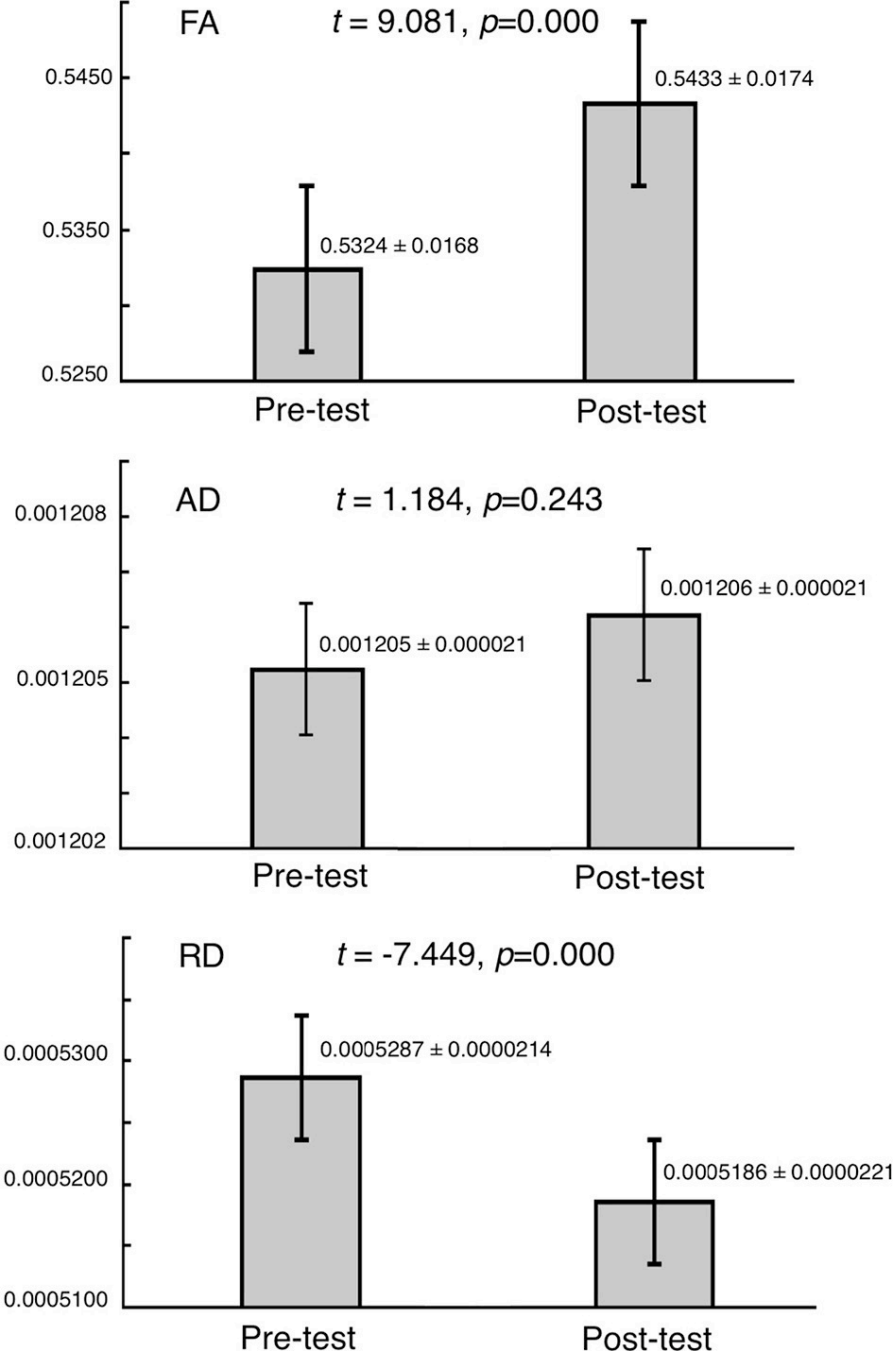
Changes in FA, AD, and RD in the right superior longitudinal fasciculus from pre- to post-training. **(Upper)** FA; **(Middle)** AD; **(Bottom)** RD.

Given the FA findings from the whole-brain analysis, we compared FA, RD, and AD values from the right superior longitudinal fasciculus in ROI analyses from pre-training to post-training in experimental group (Figure 4). Consistent with whole-brain findings, a significant increase of FA (*t* = 9.081, *p* = 0.000) was observed post-training as compared to pre-training; a significant decrease of RD (*t* = −7.499, *p* = 0.000) was also observed. No change in AD (*t* = 1.184, *p* = 0.243) was observed.

## Discussion

The present study examined diffusion-weighted MRI measures before and after short-term Internet search training. First, as expected, our results showed that the short-term Internet search training was associated with increased FA values in the right superior longitudinal fasciculus in the parietal lobe.

The current findings resonate with prior findings implicating white-matter integrity in the superior longitudinal fasciculus with spatial working memory. Multiple studies have shown that white-matter measures in the superior longitudinal fasciculus are associated with spatial working memory performance in children (Vestergaard et al., 2011), adults (Shinoura et al., 2009; Walsh et al., 2011), and individuals with schizophrenia (Karlsgodt et al., 2008; Szeszko et al., 2008), major depressive disorder (Zou et al., 2008) and attention deficit hyperactivity disorder (Hamilton et al., 2008). The training-related increase in FA in the right superior longitudinal fasciculus in this study suggests that short-term Internet search training may enhance subjects' spatial working memory performance. This result is consistent with Sparrow et al.'s finding that Internet search training may promote knowing more regarding the location of knowledge, but not necessarily knowing information *per se* (Sparrow et al., 2011). In addition, the negative correlation between FA changes in the superior longitudinal fasciculus and the changes in response time suggests that the increased FA is associated with better behavioral performance. Together, the findings suggest that short-term Internet search training may enhance spatial working memory performance through white-matter changes in the superior longitudinal fasciculus.

Second, DTI metrics have been related to axonal integrity and myelination; however, the FA values cannot distinguish whether these changes may have been caused by myelination or axonal changes (Boretius et al., 2012). To explore this question, we examined AD and RD values in the right superior longitudinal fasciculus. As mentioned above, decreased RD has been related to increased myelination (Bennett et al., 2010; Kumar et al., 2012; Tang et al., 2012; Hill, 2013). Thus, the finding of a negative correlation between FA and RD is suggestive of increased myelination in the superior longitudinal fasciculus following Internet search training.

Concurrent increases in FA increases and decreases in RD have been found in multiple prior studies, including those investigating effects of reading (Keller and Just, 2009), working memory training (Takeuchi et al., 2010; Engvig et al., 2012), and abacus training (Hu et al., 2011). Myelination has been found to be modifiable by experience, and it affects information processing by regulating the velocity and synchrony of impulse conduction between distant cortical regions (Fields, 2008, 2010). This pattern (FA↑/RD↓) has been related in corresponding brain structures to increased performance, often following training or short-/long-term practicing of one behavior. On the contrary, different pattern (FA↑/RD↓) has been observed in neurological/psychiatric diseases, such as Wallerian degeneration (Liu et al., 2013), Alzheimer's disease and mild cognitive impairment (Amlien and Fjell, 2014) and aging (de Groot et al., 2015), which are usually combined with decreased performance in related brain structures. Together, these results suggest that changes in white-matter integrity in the superior longitudinal fasciculus may result from increased myelination, and this process may promote better behavioral performance.

All these explanations are based on the role of some specific brain structures, as there is no task in DTI scan and no significant correlations were found between brain features and the behavioral responses (Supplementary Figure 1). Thus, alternative explanations might also exist, such as the brain structure changes in current study might also caused by other online activities, such as gaming. As it is impossible to control their activities for about a week's time, we cannot exclude these extra variables completely. Thus, we did not disturb their daily lives, they just act as what they usually do. The only extra activities we brought them are the 1.5 h internet search using. Happily, there is no difference in the control group in post-pre tests, which could exclude the effect from other variables in some degree.

Consistent with our priori hypothesis, the current study detected the changes in memory related brain structures (superior tempora gyrus) in post-training group comparison. However, we did not observe the similar feature in post-pre comparison in different groups. Which made us hard to explain which process caused the difference here. As we have limited subjects number in control group, we paid more attention to the comparisons between post- and pre- training in experimental groups in current study. Further studies should be performed to explore this issue.

## Limitations

Given financial limitations, fewer participants were included in the control group (16 valid subjects), leading to imbalances in subject number in the experimental and control groups. Which might affect the final results. Second, as the prevalence of Internet search using, all subjects were familiar with this activity in current study, which might also disturb the training effect. Third, we did not control subjects' behaviors outside the lab during these 6 days, there might also different degrees of search using outside the lab time. This could also disturb the final results. Fourth, we collected only one b0 in collecting DTI data, the results might be improved if we use more b0 images in data collecting and analyzing.

## Conclusions

Taken together, the current results suggest that short-term Internet search training enhances spatial working memory performance by increasing FA and reducing RD in the right superior longitudinal fasciculus. These findings suggest that the Internet search training enhanced white-matter integrity by increasing myelination in the superior longitudinal fasciculus.

## Ethics statement

All procedures performed in studies involving human participants were in accordance with the ethical standards of the institutional and/or national research committee and with the 1964 Helsinki declaration and its later amendments or comparable ethical standards. Informed consent was obtained from all individual participants included in the study.

## Author contributions

GD designed the research, revised and improved the manuscript. HL analyzed the data and prepared the figures. MP discussed the results, advised on interpretation, and contributed to the final draft of the manuscript. All authors contributed to and had approved the final manuscript.

### Conflict of interest statement

The authors declare that the research was conducted in the absence of any commercial or financial relationships that could be construed as a potential conflict of interest.
