# Supplementary material for: Short-Term Internet-Search Training Is Associated with Increased Fractional Anisotropy in the Superior Longitudinal Fasciculus in the Parietal Lobe
Source: Front Neurosci. 2017 Jun 29;11:372. doi: 10.3389/fnins.2017.00372 (PMC5489597; doi:10.3389/fnins.2017.00372)
Supplement: Supplementary file 1 [file Image1.PDF]

Supplementary material to:

Short-term Internet-search training is associated with increased fractional anisotropy in the superior longitudinal fasciculus in the parietal lobe

Correlation analysis

We calculated the correlation between FA changes and response-time (RT) changes when performing a 'remember-recall' task from pre-training to post-training (Besides the DTI scan, participants were also finished a 'remember-recall' task at the same time, which was reported in another manuscript (Dong, Potenza, 2016). A negative correlation was found between these two factors (r=-0.222, p=0.076), although it did not reach statistical significance (Supplementary Figure 1).

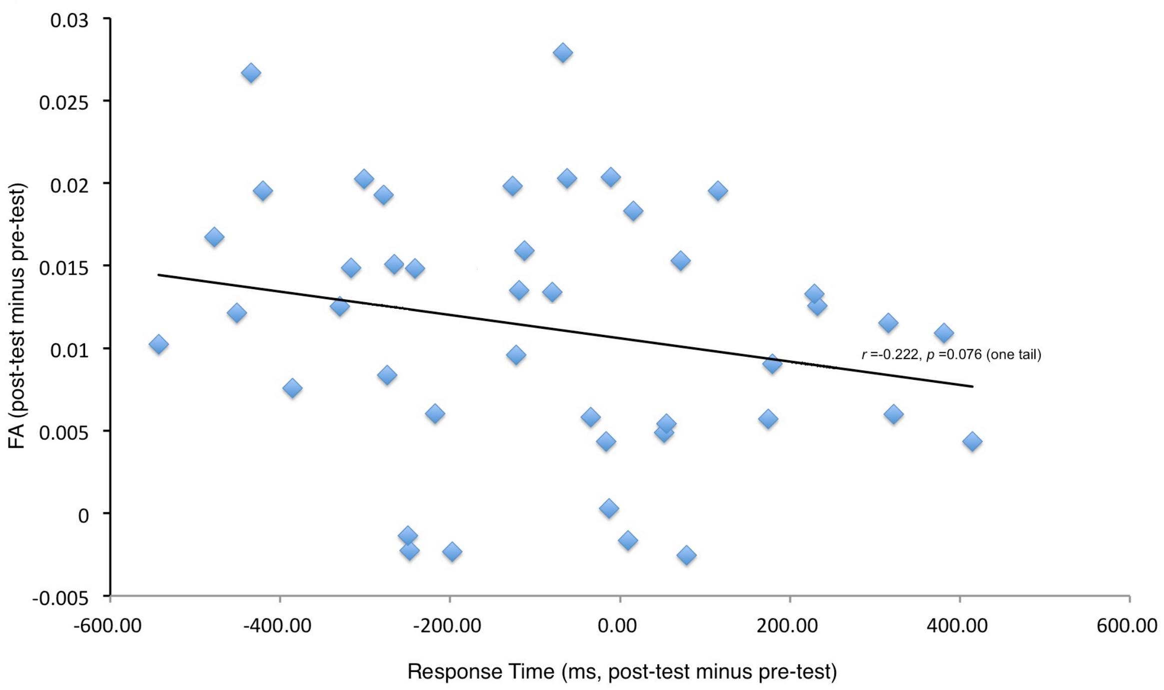

Supplementary figure 1. Correlation between response time changes in a remember-recall task and FA changes in the right superior longitudinal fasciculus from pre- to post-training.
